# Olive Mill Wastewater Inhibits Growth and Proliferation of Cisplatin- and Gemcitabine-Resistant Bladder Cancer Cells In Vitro by Down-Regulating the Akt/mTOR-Signaling Pathway

**DOI:** 10.3390/nu14020369

**Published:** 2022-01-15

**Authors:** Jochen Rutz, Sebastian Maxeiner, Eva Juengel, Felix K.-H. Chun, Igor Tsaur, Roman A. Blaheta

**Affiliations:** 1 Department of Urology, Goethe-University, 60590 Frankfurt am Main, Germany; Jochen.Rutz@kgu.de (J.R.); sebastian.maxeiner@kgu.de (S.M.); Felix.Chun@kgu.de (F.K.-H.C.); 2 Department of Urology and Pediatric Urology, University Medicine Mainz, 55131 Mainz, Germany; Eva.Juengel@unimedizin-mainz.de (E.J.); Prof.Dr.med.Igor.Tsaur@unimedizin-mainz.de (I.T.)

**Keywords:** olive mill wastewater (OMWW), bladder cancer, tumor growth, Akt-mTOR signaling, CDK-cyclin axis

## Abstract

Bladder cancer patients whose tumors develop resistance to cisplatin-based chemotherapy often turn to natural, plant-derived products. Beneficial effects have been particularly ascribed to polyphenols, although their therapeutic relevance when resistance has developed is not clear. The present study evaluated the anti-tumor potential of polyphenol-rich olive mill wastewater (OMWW) on chemo-sensitive and cisplatin- and gemcitabine-resistant T24, RT112, and TCCSUP bladder cancer cells in vitro. The cells were treated with different dilutions of OMWW, and tumor growth and clone formation were evaluated. Possible mechanisms of action were investigated by evaluating cell cycle phases and cell cycle-regulating proteins. OMWW profoundly inhibited the growth and proliferation of chemo-sensitive as well as gemcitabine- and cisplatin-resistant bladder cancer cells. Depending on the cell line and on gemcitabine- or cisplatin-resistance, OMWW induced cell cycle arrest at different phases. These differing phase arrests were accompanied by differing alterations in the CDK-cyclin axis. Considerable suppression of the Akt-mTOR pathway by OMWW was observed in all three cell lines. Since OMWW blocks the cell cycle through the manipulation of the cyclin-CDK axis and the deactivation of Akt-mTOR signaling, OMWW could become relevant in supporting bladder cancer therapy.

## 1. Introduction

Bladder cancer is the most common tumor of the urinary system worldwide [1], with approximately 550,000 diagnosed cases and more than 200,000 deaths every year [2]. The urothelial carcinoma (syn. transitional cell carcinoma) represents the predominant histologic type in the United States and Western Europe, accounting for approximately 90% of all bladder cancers [3]. Treatment of choice for non-muscle-invasive bladder cancer includes transurethral resection combined with follow-up intravesical chemotherapy or Bacillus Calmette–Guerin (BCG) immunotherapy [4]. Despite this, recurrence is high, with a 5-year progression rate of up to 45% [5].

Once metastasized, the tumor is difficult to treat. EAU Guidelines recommend first-line treatment with the MVAC (methotrexate, vinblastine, doxorubicin, cisplatin) or GC (gemcitabine, cisplatin) scheme [3]. These therapeutic approaches remain palliative, and overall survival is similar, with a median survival of 14.0 months for GC and 15.2 months for MVAC [6]. Innate and adaptive resistance towards chemotherapeutic drugs are the main reasons for therapeutic failure [7,8]. Once resistance has developed, ongoing options are limited. More than 200 appurtenant clinical trials are in progress, testifying to the urgency to improve current treatment protocols to prevent or delay drug non-responsiveness [9]. 

Dissatisfaction with conventional tumor treatment along with severe side effects under MVAC/GC drives many cancer patients to complementary and alternative medicine (CAM). Particularly the oral use of natural herbs and plant compounds has gained high interest [10], with the hope of strengthening the immune system, reducing unwanted therapeutic side effects and dynamically fighting the disease [11,12]. In fact, clinical studies indicate that integrating plant-derived compounds into conventional anti-tumor protocols may improve successful bladder cancer treatment [13]. 

Polyphenols from natural sources have been shown to act antiproliferatively on bladder cancer cells [14]. In recent years, evidence has been provided that the consumption of polyphenol-rich olive oil, as an important element of the Mediterranean diet, may not only prevent oncogenesis [15] but may also improve the function of the immune system and the efficacy of cancer treatment [16]. The present study was designed to explore the potential of polyphenol-rich olive mill wastewater (OMWW) in suppressing the growth and proliferation of bladder cancer cells with acquired resistance towards gemcitabine and cisplatin. OMWW is generated during olive oil production, and chemical characterization of OMWW extracts reveal enrichment with soluble polyphenols, of which hydroxytyrosol (HT) is the most bioactive component [17]. Besides antioxidant actions of OMWW verified in healthy volunteers [18], OMWW has been shown to suppress angiogenesis in a prostate cancer cell model [19] and to down-regulate growth and invasive properties of lung cancer cells [20]. The relevance of OMWW for drug-resistant tumor cells has not yet been explored. Evidence is provided here that OMWW inhibits proliferation of cisplatin and gemcitabine resistant cells in a panel of bladder cancer cell lines by acting on the Cyclin-CDK-axis and the Akt-mTOR signaling cascade.

## 2. Materials and Methods

### 2.1. Cell Culture

RT112, T24 (both: ATCC/LGC Promochem GmbH, Wesel, Germany), and TCCSupp (DSMZ, Braunschweig, Germany) bladder carcinoma cells were grown and cultured in Isocove’s Modified Dulbecco’s Medium (IMDM; Gibco/Invitrogen, Karlsruhe, Germany) supplemented with 10% fetal calf serum (FCS), 2% glutamax, and 1% penicillin/streptomycin (all: Gibco/Invitrogen) in a humidified, 5% CO_2_ incubator. RT112 represents an invasive (pathological stage T2) moderately differentiated (grade 2/3) model of human bladder cancer. T24 is derived from a poorly differentiated (grade 3) bladder carcinoma, whereas TCCSupp were isolated from a grade 4 transitional cell carcinoma. Subcultures from passages 7–24 were selected for experimental use. 

### 2.2. Resistance Induction

Cisplatin and gemcitabine (both provided by Hexal, Holzkirchen, Germany) were dissolved in DMSO at 1 mg/mL (cisplatin) or 20 µg/mL (gemcitabine) and stored at room temperature (cisplatin) or at 4 °C (gemcitabine). Resistance was induced by exposing parental cells to the drugs starting at 0.125 µg/mL (cisplatin) or 1.25 ng/mL (gemcitabine) and increasing stepwise to 1 µg/mL (cisplatin) or 10 ng/mL (gemcitabine-TCCSUP), or 20 ng/mL (gemcitabine-T24, RT112). This process took 3 to 6 months. To verify resistance, tumor cells were incubated for three days with cisplatin or gemcitabine free medium (figures are related to a 6-month drug pre-incubation). Subsequently, different drug concentrations (0.125 µg/mL–4 µg/mL cisplatin or 1.25 ng/mL–40 ng/mL gemcitabine) were applied to the drug-resistant and drug-sensitive cells, and cell cultures were immediately subjected to the MTT assay for 24, 48, and 72 h as described below. Controls received cell culture medium alone. Cell lines were defined as resistant when their response to drug treatment was significantly reduced compared to the response of the sensitive cell lines. Possible compound associated toxicity was determined by trypan blue (Gibco/Invitrogen). Once drug resistance had been achieved, all further investigation was carried out by comparing drug-sensitive to drug-resistant cells permanently exposed to 1 µg/mL cisplatin, 10 ng/mL gemcitabine (TCCSupp), or 20 ng/mL gemcitabine (RT112, T24).

### 2.3. Drugs and Chemicals

OMWW (OliPhenolia comp. inject) was provided by the Burg Apotheke (Königstein, Germany) and diluted in cell culture medium at 1:125–1:1000 for final use. 3,4-Dihydroxyphenylethanol (hydroxytyrosol, HT) was purchased from Cayman Chemicals (Ann Arbor, MI, USA) and used as the reference polyphenol. HT was dissolved in ethanol (EtOH) and then diluted 1:1000–1:10000 in cell culture medium. Cell culture medium with EtOH alone (1:1000) was used as a control. The influence of HT on the tumor cells was then determined with the MTT assay (see below). 

### 2.4. Cell Growth

Cell growth was assessed using the 3-(4,5-dimethylthiazol-2-yl)-2,5-diphenyltetrazolium bromide (MTT) dye reduction assay (Roche Diagnostics, Penzberg, Germany). Bladder cancer cells (50 μL, 1 × 10^5^ cells/mL, treated with OMWW diluted at 1:125–1:1000, versus non-treated) were seeded onto 96-well tissue culture plates. After 24, 48, and 72 h, 10 μL MTT (0.5 mg/mL) were added for an additional 4 h. Cells were then lysed in a buffer containing 10% SDS in 0.01 M HCl. The plates were incubated overnight at 37 °C, 5% CO_2_. Absorbance at 550 nm was determined for each well using a microplate enzyme-linked immunosorbent assay (ELISA; Tecan Infinite M200, Männedorf, Switzerland) reader. Each experiment was conducted in triplicate. After subtracting background absorbance, results were expressed as mean cell numbers. Efficacy of treatment was then calculated based on control values set to 100%. 

### 2.5. Apoptosis

To evaluate whether tumor cell growth was impaired or reduced due to apoptosis, the expression of annexin V/propidium iodide (PI) was evaluated using the annexin V-FITC Apoptosis Detection kit (BD Pharmingen, Heidelberg, Germany). In brief, tumor cells were washed twice with PBS and then incubated with 5 μL of annexin V-FITC and 5 μL of PI in the dark for 15 min at room temperature. Cells were analyzed by flow cytometry using FACScalibur (BD Biosciences, Heidelberg, Germany). The percentage of apoptotic (early and late), necrotic, and vital cells in each quadrant was calculated using CellQuest software (BD Biosciences).

### 2.6. Clonogenic Growth

One thousand single bladder cancer cells (treated with dilutions of 1:1000–1:250 OMWW, versus non-treated) were transferred to 6-well plates. Following 5 to 10 days of incubation without medium change, cell colonies were fixed and counted. Clones of at least 50 cells were counted as one colony. 

### 2.7. Cell Cycle Analysis

Cell cycle analysis was carried out with subconfluent tumor cells after 24 h cultivation with or without a dilution of OMWW. Tumor cell populations were stained with propidium iodide, using a Cycle TEST PLUS DNA Reagent Kit (BD Biosciences, Heidelberg, Germany) and then subjected to flow cytometry with a FACScan flow cytometer (BD Biosciences). Ten thousand events were collected for each sample. Data acquisition was carried out using Cell-Quest software, and cell cycle distribution was calculated using the ModFit software (BD Biosciences). The number of gated cells in the G1, G2/M, or S-phase was presented as a%.

### 2.8. Western Blot Analysis

To investigate the protein expression of cell cycle-regulating proteins in all cell lines (treated with OMWW 1:125, versus non-treated), tumor cell lysates were applied to a 7–12% polyacrylamide gel (depending on the protein size) and electrophoresed for 90 min at 100 V. The protein was then transferred to nitrocellulose membranes (1 h, 100 V). After blocking with nonfat dry milk for 1 h, the membranes were incubated overnight with monoclonal antibodies directed against the cell cycle proteins: CDK1/Cdc2 (IgG1, clone 1), pCDK1/Cdc2 (IgG1, clone 44/CDK1/Cdc2 (pY15)), CDK2 (IgG2a, clone 55), Cyclin A (IgG1, clone 25), Cyclin B (IgG1, clone 18; all: BD Pharmingen), pCDK2 (Thr160, New England Biolabs, Frankfurt, Germany). The mechanistic target of rapamycin (mTOR) pathway was investigated using the following monoclonal antibodies: Raptor (clone 24C12), pRaptor (clone Ser 792), Rictor (clone D16H9), pRictor (clone Thr1135, clone D30A3), mTOR (clone 7C10), pmTOR (clone D9C2; all: New England Biolabs), PKBα/Akt (IgG1 clone 55), anti phospho Akt (pAkt; IgG1, Ser472/Ser473, clone 104A282; both: BD Pharmingen). HRP-conjugated goat anti-mouse IgG and HRP-conjugated goat anti-rabbit IgG (both: 1:5000; Upstate Biotechnology, Lake Placid, NY, USA) served as the secondary antibody. The membranes were briefly incubated with an ECL detection reagent (ECL; Amersham/GE Healthcare, München, Germany) to visualize the proteins and then analyzed by the Fusion FX7 system (Peqlab, Erlangen, Germany). β-Actin (1:1000; clone AC-15; Sigma-Aldrich, Taufenkirchen, Germany) served as the internal control. Pixel density analysis of the protein bands (both total and phosphorylated) was achieved by calculating the ratio of protein intensity/β-actin intensity (GIMP 2.8 software, www.gimp.org, accessed on 9 October 2021).

### 2.9. Statistics

All experiments were performed three to six times. Statistical significance was calculated with the Wilcoxon–Mann-Whitney U test or with ANOVA along with the Dunnett’s test. Differences were considered statistically significant at a *p*-value less than 0.05.

## 3. Results

### 3.1. Resistance Induction

**Figure 1:**
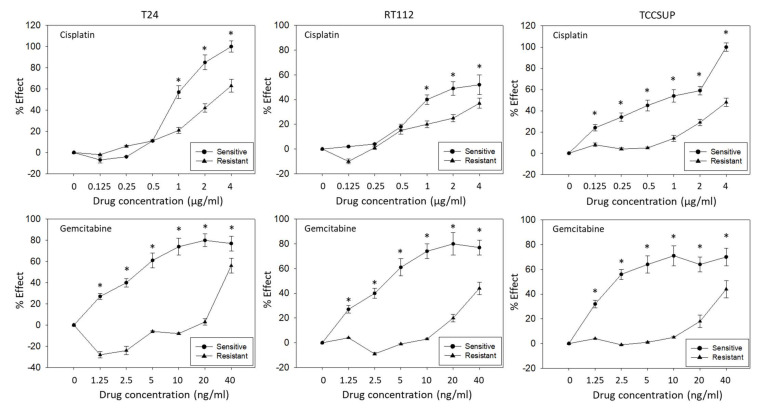
Growth blocking efficacy of increasing concentrations of cisplatin (upper graphs) and gemcitabine (lower graphs) on sensitive versus resistant T24, RT112, and TCCSUP bladder cancer cell lines. % Effect refers to a reduction of the 24–72 h increase in tumor cell number compared to untreated cells. (0% effect = no reduction, 100% effect = complete loss.) Error bars indicate standard deviation (SD). * indicates significant difference to the corresponding control, *n* = 5.

T24, RT112, and TCCSUP cells were treated with increasing doses of cisplatin or gemcitabine to induce resistance. Figure 1 demonstrate the dose–response relationship of parental (sensitive) and resistant sublines. Concentrations of 1–4 µg/mL cisplatin caused a significant reduction of the drug’s efficacy on tumor growth of resistant T24 and RT112 cells, compared to the treatment of the cisplatin-sensitive cells. In TCCSUP cells, differences were already seen at 0.125 µg/mL cisplatin (Figure 1). The response of T24, RT112, and TCCSUP cells to gemcitabine in a concentration range of 0.125–40 ng/mL also differed significantly between resistant and sensitive cells. Application of the trypan blue assay showed that drug treatment induced no toxic effects.

### 3.2. OMWW Blocks Tumor Cell Growth

**Figure 2:**
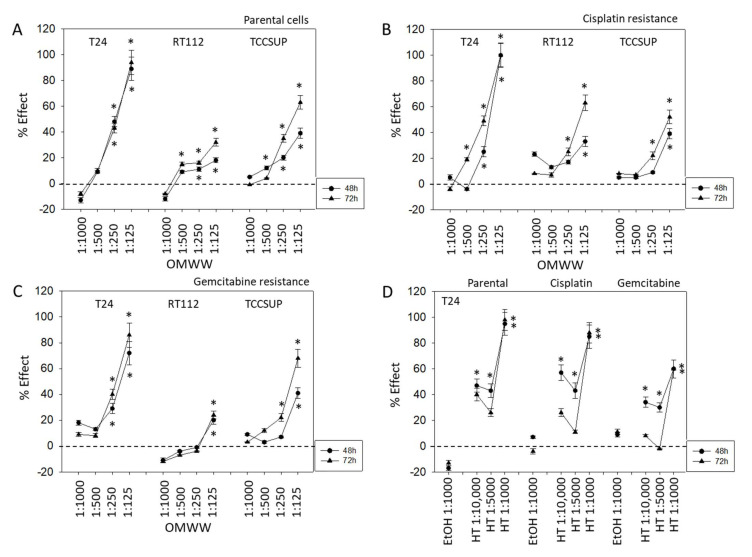
Influence of 48 and 72 h OMWW incubation on the growth of parental (**A**), cisplatin- (**B**) and gemcitabine-resistant (**C**) T24, RT112, and TCCSUP bladder cancer cell lines. Efficacy is expressed as a percentage compared to controls (without OMWW) set to zero. (**D**) Efficacy of different hydroxytyrosol (HT) concentrations or ethanol in diminishing the number of T24 tumor cells (parental, cisplatin-, and gemcitabine-resistant), compared to the control (EtOH 1:1000) set to zero. Error bars indicate standard deviation (SD), *n* = 5. * indicates significant difference to the corresponding control.

OMWW significantly blocked cell growth in all three cell lines (Figure 2). Suppressive effects were seen on parental, cisplatin, and gemcitabine resistant tumor cells. However, the percentage effect depended on the cell line used and the resistance status. About 48 h after adding OMWW, a distinct growth blocking effect was seen when parental cells were treated with OMWW at a dilution of 1:250 or 1:125. TCCSUP even responded to 1:500 OMWW (Figure 2A). The strongest effects were evoked on RT112 and TCCSUP after 72 h OMWW incubation, whereas no time-dependent differences were observed in T24 cell cultures. Here effects seen after 48 h OMWW incubation were similar to those observed after 72 h. OMWW was also effective in diminishing the number of cisplatin-resistant T24, RT112, and TCCSUP cells (72 h incubation > 48 h incubation). At 72 h incubation, OMWW acted on RT112 and TCCSUP cells at a 1:250 and 1:125 dilution. Cisplatin-resistant T24 cells already responded well to 1:500 OMWW (Figure 2B). A significantly diminished cell number was also seen when gemcitabine-resistant tumor cells were exposed to OMWW. Both 1:125 and 1:250 OMWW reduced the T24 cell number after 48 and 72 h, compared to the controls, whereas the 1:250 dilution was only effective on TCCSUP after 72 h, but not after 48 h. An OMWW dilution of 1:125 was necessary to suppress RT112 growth. Higher dilutions were without effect (Figure 2C). To evaluate whether hydroxytyrosol (HT), the main component of OMWW, contributes to cell growth reduction, T24 tumor cells (sensitive and resistant) were also exposed to HT, and cell growth was analyzed. HT at 1:1000 dilution, which matches the concentration in OMWW 1:125, significantly diminished growth of parental and resistant T24, TCCSUP, and RT112 cells after 48 and 72 h incubation, each compared to the untreated controls (Figure 2D). Application of the solvent alone (ethanol 1:1000 in cell culture medium) did not induce any alteration in tumor cell growth (Figure 2D). 

### 3.3. Apoptosis Induction by OMWW

**Figure 3:**
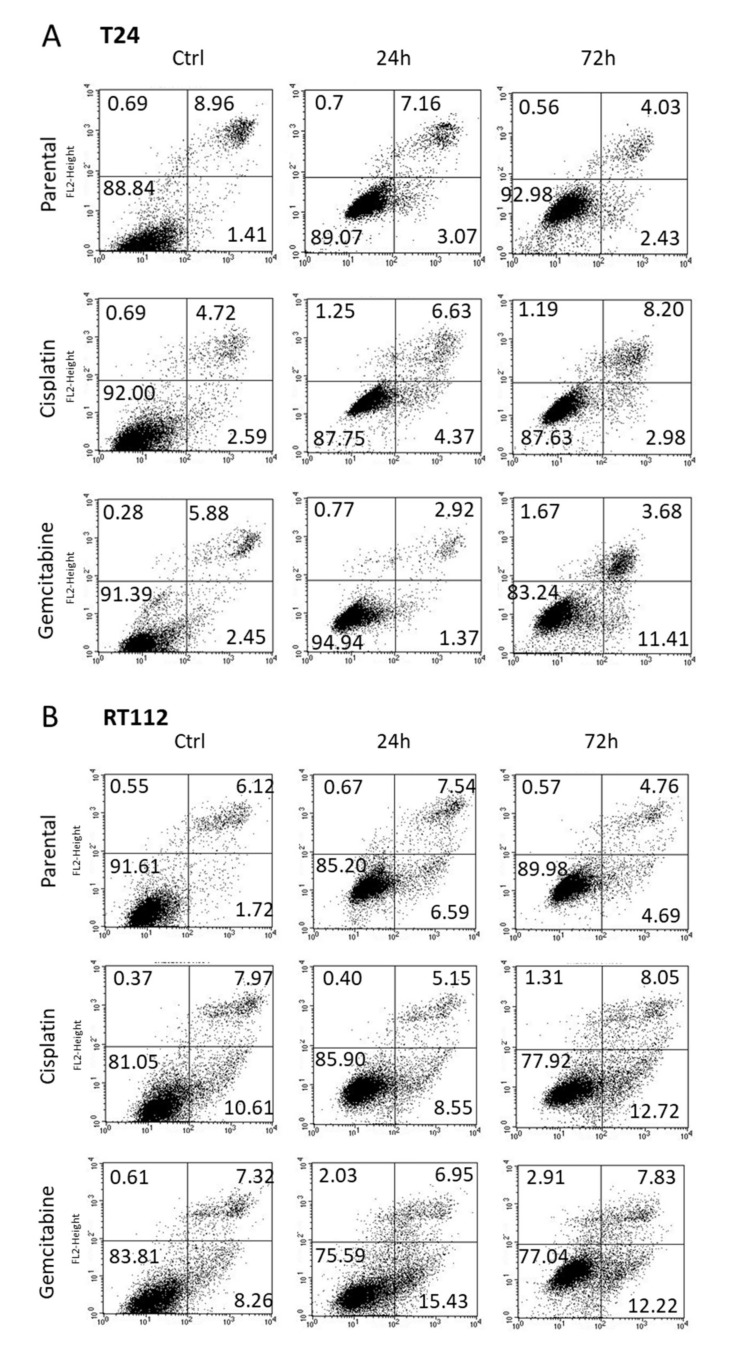
Early apoptosis, late apoptosis and necrosis of parental and resistant T24 (**A**) and RT112 (**B**) cells treated with OMWW 1:250 for 24 and 72 h. Controls remained untreated. Upper left quadrants to show the percentage of cells in necrosis, upper right quadrants percentage of cells in late apoptosis, lower right quadrants percentage of cells in early apoptosis, and lower left quadrants vital cells (one representative of 3 analyses; SDintra-assay < 10%).

Apoptosis was evaluated in T24 (Figure 3A) and RT112 (Figure 3B) cells 24 and 72 h following exposure to OMWW, diluted 1:250. Necrosis was not induced. However, OMWW caused a slight elevation of apoptotic cisplatin-resistant T24 cells after 72 h (late apoptosis) and enhanced the percentage of gemcitabine-resistant T24 cells in early apoptosis after 72 h. A moderate increase of gemcitabine-resistant RT112 cells undergoing early apoptosis was also apparent 24 h after OMWW addition. 

### 3.4. OMWW Suppresses Clonogenic Tumor Growth

**Figure 4:**
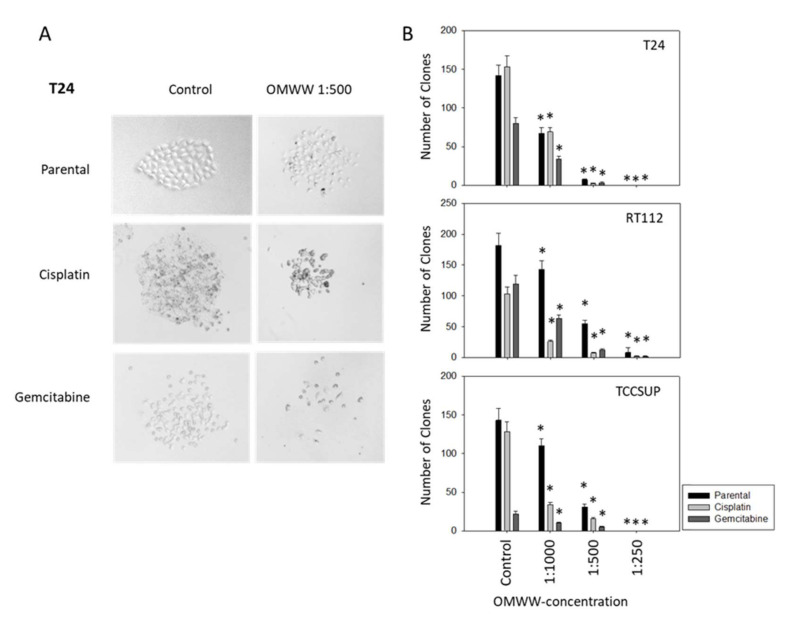
(**A**) Influence of OMWW 1:500 on clone formation in parental, cisplatin-resistant, and gemcitabine-resistant T24 cells. Controls are without OMWW. (**B**) Number of T24, RT112, and TCCSUP clones (parental, cisplatin-resistant, gemcitabine-resistant) exposed to different OMWW-concentrations. Controls are without OMWW. Error bars indicate standard deviation (SD), *n* = 3. * indicates significant difference to untreated controls.

To investigate the potential of OMWW to stop colony formation, the clonogenic growth assay was performed. The number and size of tumor cell clones were significantly diminished in a concentration-dependent manner, whereby OMWW acted on both parental and drug-resistant cell lines. Figure 4A representatively demonstrate the destruction of T24 clones following OMWW exposure (1:500 dilution). Effects on the clone number were already detected at concentrations of 1:1000 with nearly complete disintegration at an OMWW dilution of 1:250 (Figure 4B). 

### 3.5. OMWW Causes Cell Cycle Alterations

**Figure 5:**
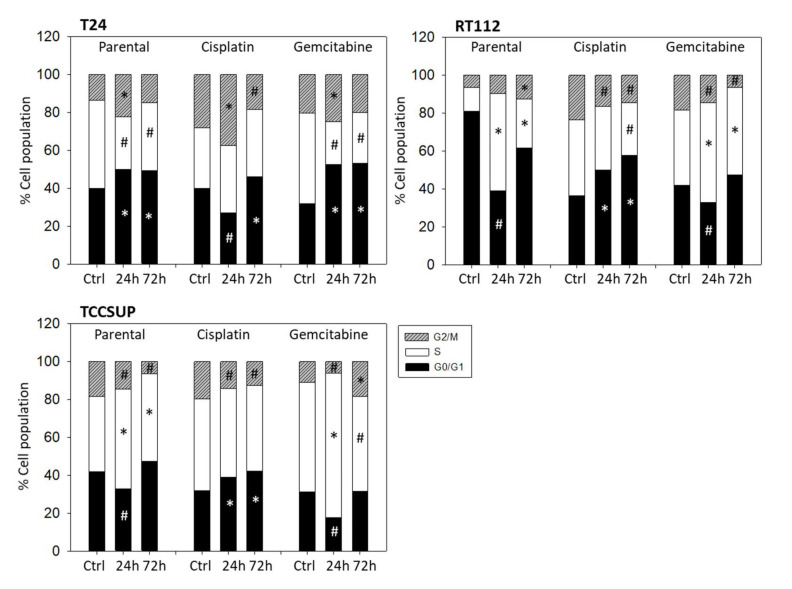
Cell cycle distribution in parental and resistant T24, RT112, and TCCSUP cells following OMWW (dilution 1:250) exposure for 24 and 72 h. Controls (Ctrl) remained untreated. One representative of three separate experiments is shown (*n* = 3). * indicates significant up-regulation, # indicates significant down-regulation, compared to the respective control.

Distinct cell cycle alterations were evoked following OMWW 1:250 application. The kind of alteration depended on the OMWW incubation time and the cell line used. In both sensitive and resistant T24 cells, the proportion of G2/M-phase cells significantly increased after 24 h (Figure 5). After 72 h, particularly in the cisplatin-sensitive cells, OMWW caused an increase in G0/G1 with a simultaneous decrease of G2/M-phase cells. The percentage of sensitive RT112 S-phase cells under OMWW was also more pronounced after 24 h, compared to the 72 h value. However, parental RT112 cells in G2/M increased time-dependently, reaching a maximum after 72 h. The opposite became evident in cisplatin- and gemcitabine-resistant RT112, where the number of G2/M-phase cells declined over time. A time-dependent decline of G2/M-phase cells was also noted in parental and cisplatin-resistant TCCSUP cells, with the highest number of cells in G0/G1 after 72 h OMWW incubation. Gemcitabine-resistant TCCSUP differed in that G2/M was diminished after 24 h OMWW incubation but enhanced after 72 h. This was associated with elevated S-phase cells after 24 h but a loss of S-phase cells after 72 h (Figure 5).

### 3.6. Evaluation of Cell Cycle Regulating Proteins

**Figure 6:**
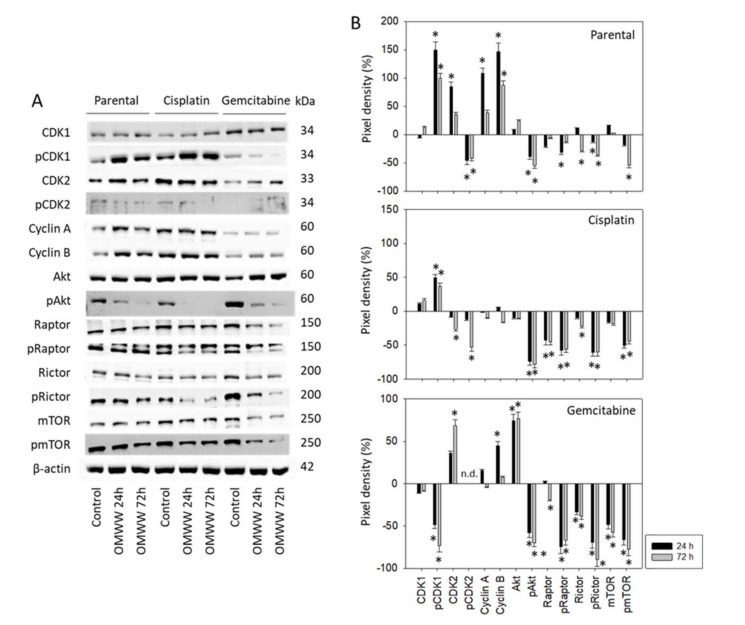
(**A**) Western blot of cell cycle and mTOR related proteins from RT112 lysates of parental and cisplatin/gemcitabine-resistant cells. Tumor cells were pretreated with OMWW (dilution 1:125) for 24 and 72 h (controls remained untreated). β-actin served as the internal control. One representative from three separate experiments. (**B**) The ratio of protein intensity/β-actin intensity expressed as a percentage of the controls, set to zero. Error bars indicate standard deviation (SD), *n* = 3. * indicates significant difference to controls.

Subsequent studies concentrated on cell cycle-regulating protein expression in parental, cisplatin-, and gemcitabine-resistant RT112 and T24 cells. OMWW induced a time-dependent increase of pCDK1, CDK2, Cyclin A and B in parental RT112 cells with maximum effects after 24 h (Figure 6A-Western blot and Figure 6B-pixel density, Supplementary Figure S1). In contrast, pCDK2 was time independently considerably diminished. Suppression of pAkt (24 h, 72 h), pRaptor (24 h), Rictor (24 h, 72 h), pRictor, and pmTOR (both 72 h) were also observed. In cisplatin-resistant RT112 cells, pCDK1 was up-regulated equally well by OMWW after 24 and 72 h, whereas both CDK2 and pCDK2 were only down-regulated after 72 h. Cyclin A and B were not altered by OMWW. Phosphorylated proteins of the Akt-mTOR axis were reduced to the same extent after 24 and 72 h. Diminished activity of the Akt-mTOR axis was also seen when gemcitabine-resistant RT112 cells were treated with OMWW (24 h, 72 h). However, contrary to the decrease of pAkt, the total Akt protein was elevated under OMWW after both 24 and 72 h. Phosphorylated pCDK1 was suppressed after 24 h and nearly lost after 72 h incubation with OMWW. Phosphorylation of CDK2 was not clearly detectable in the gemcitabine-resistant cells. 

**Figure 7:**
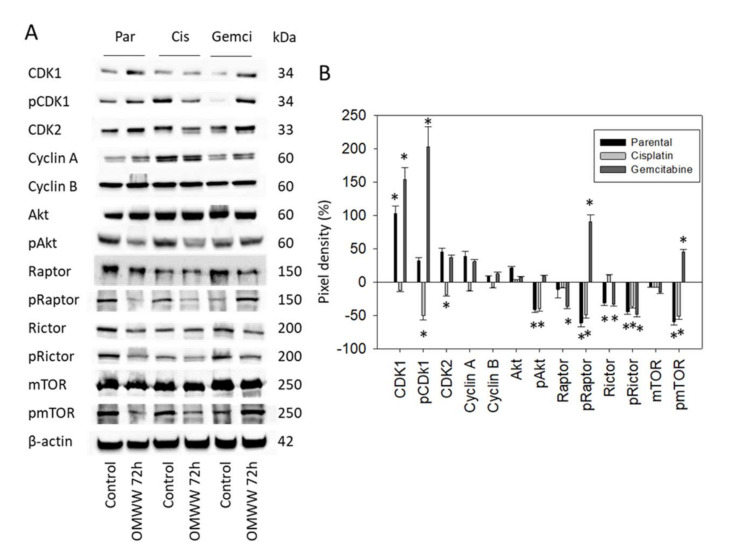
(**A**) Western blot of cell cycle and mTOR related proteins from lysates of parental and cisplatin/gemcitabine-resistant T24 cells. Tumor cells were pretreated with OMWW (dilution 1:125) for 72 h (controls remained untreated). β-actin served as the internal control. One representative from three separate experiments. (**B**) The ratio of protein intensity/β-actin intensity expressed as a percentage of the controls, set to zero. Error bars indicate standard deviation (SD), *n* = 3. * indicates significant difference to controls.

T24 cells were also evaluated following 72 h OMWW-treatment. CDK1 was enhanced in the parental and gemcitabine-resistant cells, whereas pCDK1 and CDK2 were reduced in the cisplatin-resistant subline (Figure 7A-Western blot and Figure 7B-pixel density, Supplement S1). pAkt, pRaptor, and pmTOR were all suppressed in parental and cisplatin-resistant cells. However, pRaptor and pmTOR increased in the gemcitabine-resistant subline. pRictor was found to be down-regulated in all T24 cell sublines. 

## 4. Discussion

The Mediterranean diet is associated with a lower cancer risk that has been attributed to the extensive use of olive oil. Indeed, olive oil polyphenols, with HT as the main component, have been shown to be involved in anti-carcinogenic mechanisms and pathways [21]. Novel data point to a strong proliferation suppression of four different human bladder cancer cell lines following exposure to polyphenol-rich extracts from steam-treated olives [22]. Evidence is provided here that HT-rich OMWW is highly efficient in blocking the growth of parental as well as cisplatin- and gemcitabine-resistant bladder cancer cells. Depending on the cell line and resistance to cisplatin or gemcitabine, OMWW was effective at dilutions ranging from 1:125 to 1:500. This agrees with another experimental model, where OMWW at a dilution ranging from 1:50 to 1:250 significantly blocked the growth of lung cancer cell lines [20]. The same efficacy was seen with DU145 and PC3 prostate cancer cells, whereby the growth of the prostate cancer cell line LNCaP was already blocked by an OMWW-dilution of 1:5000 [19]. The exceptional action of OMWW on LNCaP cells, observed only after 96 h incubation, was traced to a drastic increase in apoptosis, neither observed in DU145 nor PC3 prostate cancer, nor in lung or colon cancer cells [23]. Albini et al. recently showed that OMWW enhances the effect of cisplatin chemotherapy on prostate cancer xenografts [24]. In the present investigation, we found no distinct increase of apoptotic events after OMWW exposure over a time period of 72 h. Therefore, exposure to OMWW, which inhibits bladder cancer cell growth along with reducing cell clone number and size, cannot be attributed to apoptosis. 

Depending on the cell line and resistance status, cell cycle analysis revealed an inhomogeneous picture as to how OMWW time-dependently acts on tumor cells. This inconsistent response to OMWW in tumor cell lines from the same tumor type has been observed by others, as well. Baci et al. reported apoptosis regulation by OMWW differing between androgen-sensitive and androgen-resistant prostate cancer cells [19]. Following OMWW exposure Gallazzi and coworkers observed altered production of angiogenic and inflammatory mediators in different lung cancer cell lines [20]. To date, no definitive explanation for why OMWW differentially acts on cell cycling has been proposed. Evidence has been provided that membrane receptors of the integrin α and β family serve as crucial mediators of outside-in-signaling in controlling cell survival and proliferation, whereby the expression patterns and functional roles of integrins vary among tumor entities [25]. Characterization of a panel of bladder cancer cell lines has revealed different involvement of α and β integrins [26], associated with different responses to external stimuli [27]. Integrin triggered intracellular signaling is also different in resistant and non-resistant tumor cells [28]. The duration of cell manipulation differentially regulates cell signaling, as well. Rutz et al. [29] and Justin et al. [30] recently showed that tumor cells treated with the phytochemical sulforaphane were arrested at different cell cycle phases in a time-dependent manner. We did not investigate how OMWW may alter integrin-dependent signaling and how the integrin expression pattern may correlate with cell cycle blockade. Therefore, assuming that an inhomogeneous integrin expression level and activity might at least partially be responsible for the inhomogeneous influence of OMWW on cell cycling remains speculative. 

Independent of the role of integrins, we observed that OMWW influenced cell signaling processes, whereby alterations induced in the cyclin-CDK axis were different, depending on the cell line (differing tumor grades) and chemosensitivity. pCDK1, CDK2, and Cyclin B strongly increased after 24 h OMWW exposure in parental RT112 cells, which might explain the maximum increase in S-phase and decrease in G0/G1-phase cells at the 24 h time-point. In contrast, a time-dependent loss of CDK2 and pCDK2 in cisplatin-resistant RT112 cells correlated with a time-dependent elevation in G0/G1-phase cells. The number of gemcitabine-resistant S-phase cells increased to a maximum level after 24 h of OMWW exposure, whereas a maximum decrease of pCDK1 and maximum enhancement of CDK2 was noted after 72 h. Whether this effect was predominantly responsible for the G2/M-phase accumulation in this cell subline, with maximum values after 72 h exposure, can only be speculated upon. In fact, the role of CDK-cyclins in cell-cycle transition processes is multifaceted and not fully elucidated. Reducing the expression of cyclin B and increasing the expression of pCDK1 has been shown to be associated with a G2/M phase arrest in several bladder cancer cell lines [31]. This contrasts with data from Lee et al., where G2/M-arrest increased protein expression of both cyclin B and pCDK1 and decreased total CDK1 [32]. Down-regulation of CDK1 has been shown by others to contribute to an S-phase arrest in bladder cancer cells [33], although Aranha et al. pointed to dephosphorylating CDK2 as the relevant mechanism for arresting bladder cancer cells at the S/G2/M checkpoints [34]. Analysis of 32 transitional cell carcinoma specimens has confirmed the heterogeneity of cyclins and CDKs in their biological behavior [35]. In our investigation, the cell cycle arrest caused by OMWW was paralleled by distinct alterations of CDK1 and 2 expression and cyclin A and B activity. A homogenous OMWW mode of action was not apparent. Rather, OMWW effects depended on the cell line used, the chemosensitivity of the cells, and the incubation time. 

In contrast to the variable influence of OMWW on the cyclin-CDK complex, depending on the cell line, chemosensitivity, and incubation time, the Akt-mTOR signaling cascade was similarly deactivated by OMWW in chemoresistant and chemosensitive RT112 cells (except for slight time-dependent differences in the chemosensitive cells). An integrated analysis of 131 urothelial carcinomas has identified the Akt/mTOR pathway as a potential therapeutic target in 42% of these tumors [36]. Several mTOR-inhibitors have been developed and clinically approved, but all of these drugs have severe, commonly occurring side effects, and undesired feedback mechanisms evolve rapidly. This stands in contrast to the use of natural compounds whose limited cytotoxic effects and high specificity, particularly regarding plant-based polyphenols, have led to increasing attention in cancer biology [37]. The natural polyphenol, resveratrol, has been shown to slow the growth of bladder tumors in vivo and induce cell cycle arrest in vitro by inhibiting Akt/mTOR signaling, similar to the action of OMWW. Resveratrol also counteracts gemcitabine resistance in bladder cancer cells [38]. Likewise, the polyphenolic compound curcumin has been demonstrated to reverse gemcitabine resistance of bladder cancer cells, and positive effects of curcumin with cisplatin-based therapy have been shown in vivo and in vitro [39]. The strong suppression of Akt-mTOR signaling by OMWW supports the introduction of this drug into established treatment protocols. Still, notice should be taken of gemcitabine-resistant T24 cells showing a strong loss of the mTOR member Rictor (both total and phosphorylated), but an elevation of pRaptor. Whether a feedback mechanism develops or whether increased pRaptor represents an unspecific epiphenomenon is not clear. Since the total Raptor was diminished by OMWW in the gemcitabine-resistant T24 cells, we assume that this effect at least counteracts pRaptor up-regulation. It should also be noted that gemcitabine-resistant T24 cells responded to OMWW most potently in terms of growth reduction, compared to the response of gemcitabine-resistant RT112 and TCCSUOP cells. It, therefore, seems unlikely that the enhancement of pRaptor is associated with resistance development towards OMWW. 

Whether the anti-tumor effects of OMWW are exclusively based on its polyphenolic content is not clear. HT is the most abundant polyphenol present in OMWW. Our experiments point to a distinct down-regulation of T24 growth induced by HT, indicating that this molecule may be the main factor responsible for the anti-tumor potential of OMWW. Studies on lung cancer cells have revealed a stronger effect of OMWW on tumor proliferation than that observed in cells treated with HT, adjusted to the OMWW-dilution [20]. This was not observed in a colon cancer cell model where OMWW effects were similar to those exerted by comparable concentrations of HT alone [23]. Nevertheless, this issue requires further attention. In fact, other phenols found in OMWW have been shown to have antitumorigenic properties. Verbascoside has recently been reported to mitigate cell proliferation and aggressiveness of prostate cancer [40], and novel data emphasize the role of oleuropein in the reduction of cisplatin resistance in ovarian cancer [41]. The polyphenol composition in OMWW may therefore act synergistically on tumor cells.

## 5. Conclusions

We provide evidence that OMWW profoundly acts on the growth and proliferation of bladder cancer cells, both in chemo-sensitive as well as gemcitabine- and cisplatin-resistant cells. Still, evidence has not been provided here that OMWW may reverse drug resistance. Future work should specifically investigate whether OMWW counteracts resistance to cisplatin and gemcitabine in bladder cancer cells. Manipulation of the cyclin-CDK axis and de-activation of Akt-mTOR signaling is suggested to be responsible for the cell cycle blockade evoked by OMWW. We propose that OMWW might be a valuable candidate to support bladder cancer therapy. The promising in vitro results should be confirmed in vivo in a murine model. The size and weight of removed tumors should be compared with and without OMWW treatment.

## Supplementary Materials

The following are available online at https://www.mdpi.com/article/10.3390/nu14020369/s1, Figure S1: Western blot in Figure 6A and Figure 7A.
