# Supplementary figures and images for: Olive Mill Wastewater Inhibits Growth and Proliferation of Cisplatin- and Gemcitabine-Resistant Bladder Cancer Cells In Vitro by Down-Regulating the Akt/mTOR-Signaling Pathway
Source: Nutrients. 2022 Jan 15;14(2):369. doi: 10.3390/nu14020369 (PMC8778865; doi:10.3390/nu14020369)

Supplement Figure S1 Western Blot, Figure 6A

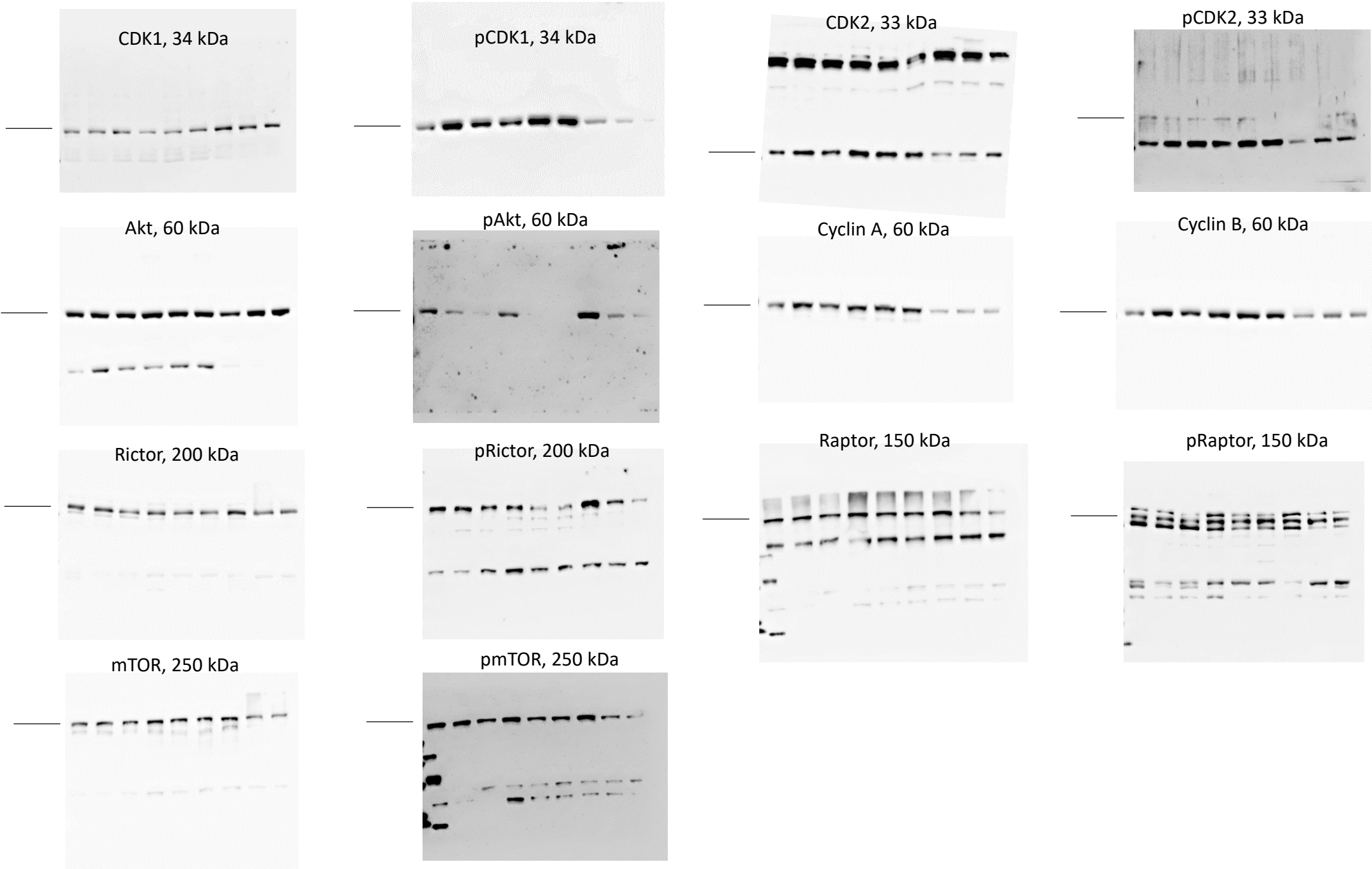

Western Blot, Figure 7A

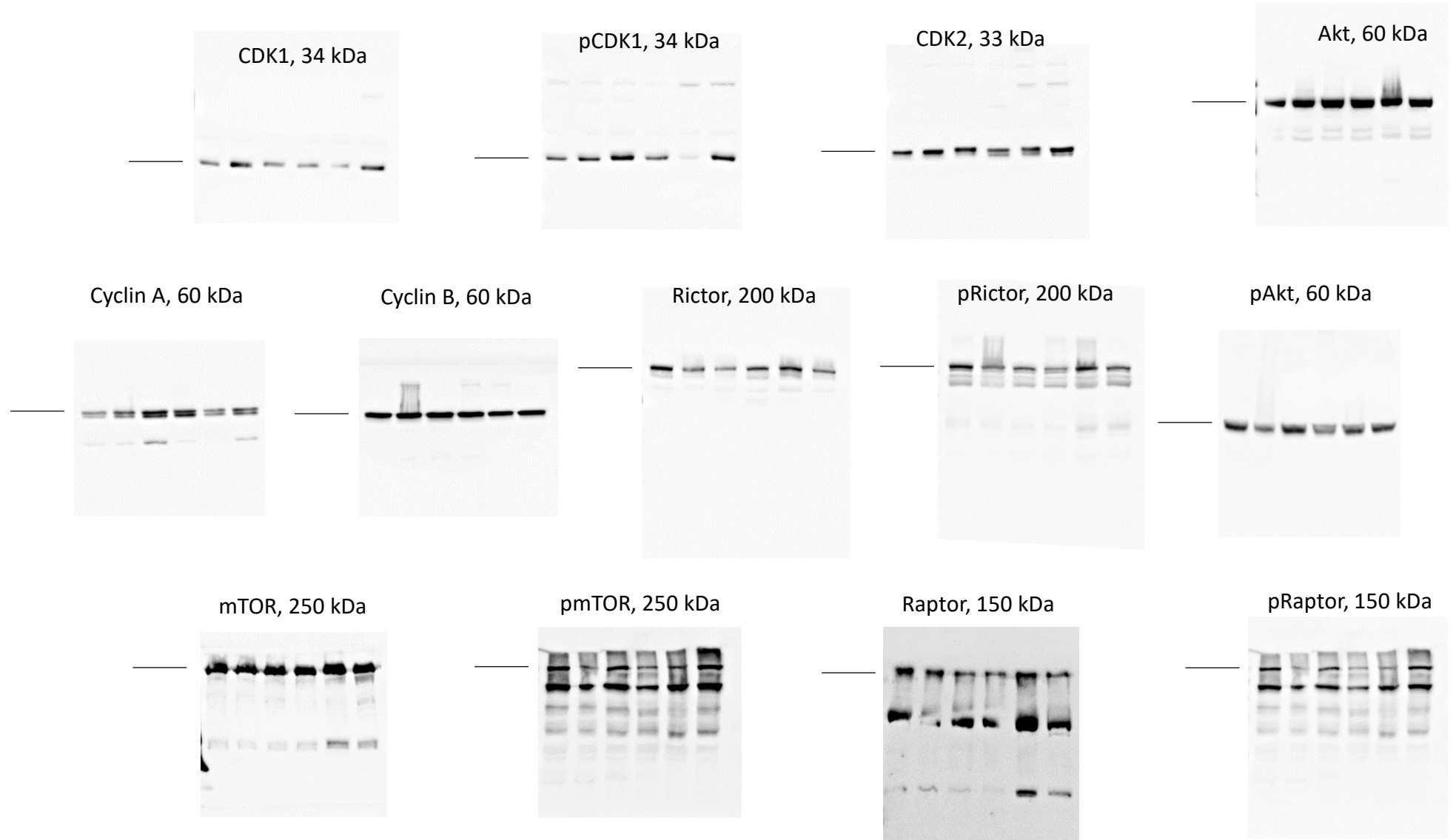

Supplement: Supplementary file 1 [file nutrients-14-00369-s001.zip › nutrients-1473658-Supplementary.pdf]
